# Association of Epidermal Growth Factor Receptor Mutations with Metastatic Presentations in Non-Small Cell Lung Cancer

**DOI:** 10.5402/2011/756265

**Published:** 2011-04-27

**Authors:** Im Il Na, Jong Heon Park, Du Hwan Choe, Jin Kyung Lee, Jae Soo Koh

**Affiliations:** ^1^Department of Internal Medicine, Korea Cancer Center Hospital, Korea Institute of Radiological & Medical Sciences, 215-4, Gongneung-dong, Nowon-gu, Seoul 139-706, Republic of Korea; ^2^Department of Radiology, Korea Cancer Center Hospital, Korea Institute of Radiological & Medical Sciences, Seoul 139-706, Republic of Korea; ^3^Department of Laboratory Medicine, Korea Cancer Center Hospital, Korea Institute of Radiological & Medical Sciences, Seoul 139-706, Republic of Korea; ^4^Department of Pathology, Korea Cancer Center Hospital, Korea Institute of Radiological & Medical Sciences, Seoul 139-706, Republic of Korea

## Abstract

We performed this retrospective study to assess the association of *epidermal growth factor receptor* (EGFR) with metastatic presentations in advanced non-small cell lung cancer (NSCLC). The data from 125 patients with stage III or IV NSCLC were analyzed. We detected EGFR mutations in 36 NSCLC patients. EGFR mutations were predominant in never-smokers (*P* < .001), patients with adenocarcinomas (*P* < .001), and female patients (*P* < .001). When the metastatic sites were analyzed, pleural metastases were associated with a high incidence of EGFR mutations (*P* = .028). Particularly, pleural metastases with minimal effusion (PMME) were associated with EGFR mutational status (*P* = .001). Patients with N3 lesions were less likely to harbor EGFR mutations (*P* = .033). On multivariate analysis, N3 lesions (*P* = .017) and PMME (*P* < .001) remained significant factors for EGFR mutations. EGFR mutations may be associated with different presentations of pleural and N3 nodal metastases.

## 1. Introduction

In terms of new cases, lung cancer is one of the most common cancers worldwide [1, 2], and the most frequent type is non-small cell lung cancer (NSCLC). Patients with early stage NSCLC have long-term survival with surgical resection; however, the majority of patients present with advanced stage NSCLC (III or IV) have a dismal prognosis with disease progression [3, 4]. 

To improve clinical outcome in patients with NSCLC, tyrosine kinase inhibitors (TKIs), such as gefitinib or erlotinib, were introduced. With respect to TKI responsiveness, recent molecular studies have shown that mutations in the *epidermal growth factor receptor* (EGFR) can predict outcomes [5]. It has been demonstrated that EGFR mutations are frequently found in females, patients with adenocarcinomas, and never-smokers [6, 7]. Researchers have also reported that most EGFR mutations consist of exon 19 deletions and exon 21 L858R substitutions [6, 7]. 

Metastases to the pleura and lymph nodes at the time of presentation are common and confer a poor prognosis in patients with stage III or IV NSCLC [8]. In patients with adenocarcinomas, the predominant histology of EGFR-mutant tumors [6], pleural metastases are a frequent finding [9]. Recently, genetic studies of other solid tumors have suggested that there are preferential metastatic sites according to gene expression [10, 11]. An animal model of lung cancer has also shown different patterns of pleural and nodal metastases according to genetic expression related to angiogenesis and lymphangiogenesis [12]. Interestingly, a high detection rate of EGFR mutations (approximately 70%) has been reported in malignant pleural effusions of pulmonary adenocarcinoma [13]. However, associations between metastatic presentations and EGFR mutations have not been fully evaluated in patients with advanced NSCLC. 

Clinical features may help physicians select patients likely to benefit from treatment with TKIs, while genetic tests have several limitations, such as insufficient material and a time-consuming process. In addition, evaluation of different clinical presentations according to EGFR mutations may add new insight for further therapy. We performed this retrospective study to identify possible associations between metastatic presentations and EGFR mutations.

## 2. Materials and Methods

### 2.1. Patients

**Figure 1:**
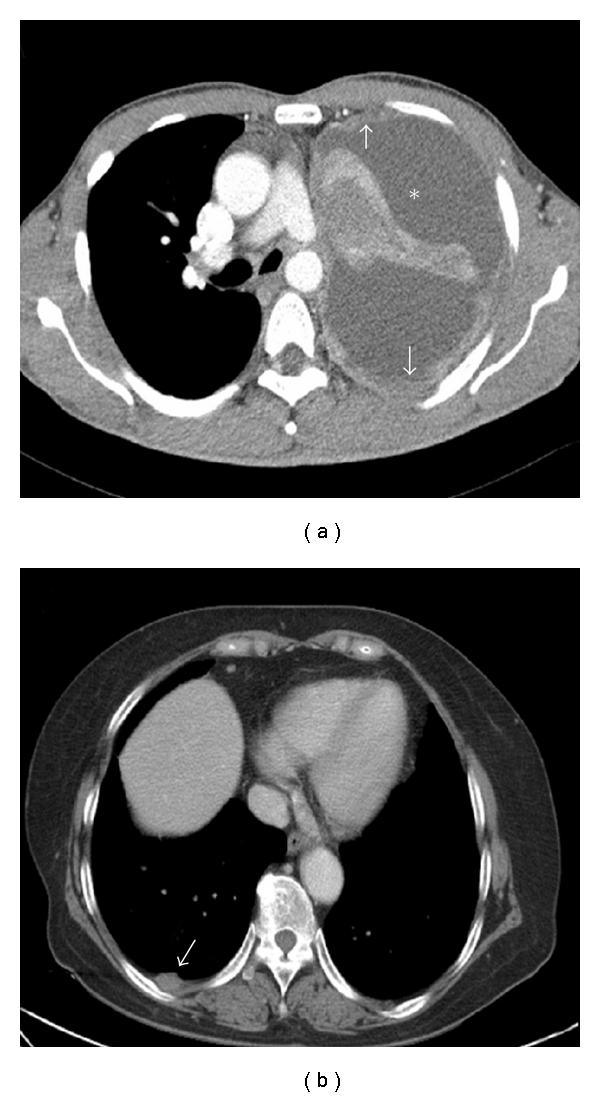
Pleural metastases with large amount of effusion (∗) and diffuse pleural thickening (arrows) in a patient with wild-type EGFR. (a) Focal nodularity (arrows) without pleural effusion in a patient with L858R substitution in exon 21 (b).

We initially identified patients who had documented results for EGFR mutational status from the NSCLC pathology database of the Korea Cancer Center Hospital (Seoul, Republic of Korea) between March 2007 and June 2010. Informed consent for genetic tests was also required. Among the initially identified patients, those with stage III or stage IV NSCLC were included using new criteria [8]. Four patients with histories of other malignancies, except thyroid cancer, were excluded. One hundred twenty-five patients were included. T and N stages were decided based on findings of computed tomography (CT). Pleural metastases were considered positive based on cytologic examinations or CT scans revealing the following criteria: (1) massive pleural effusion with or without pleural thickening, (2) circumferential thickening, (3) focal and/or diffuse nodularity of the pleura, (4) parietal pleural thickening >1 cm, and (5) mediastinal pleural thickening [14–16]. Pleural metastases were categorized into pleural metastases with minimal effusion (PMME) and non-PMME. We defined PMME as pleural metastases without effusion or those not detected on chest radiography but only on CT (Figure 1). Two thoracic radiologists (DHC and JHP) reviewed the CT images. Decisions on CT findings were reached by consensus. Regional lymph nodes larger than 1 cm in the short axis on transaxial CT images were considered positive. Metastases to brain and bone were determined using previously described criteria [17]. The Institutional Review Board of the Korea Cancer Center Hospital approved this study.

### 2.2. EGFR Genotyping

Genomic DNA was extracted from 114 paraffin-embedded tissues, as described previously in [18]. In eleven patients, methanol-fixed cytologic specimens were used for DNA extraction [19]. The EGFR mutations of 52 patients were analyzed by direct sequencing [18]. Pyrosequencing was performed in 73 patients as follows: DNA was amplified with PCR primer sets, and one strand of each set was bound to biotin at the 5′ end (primer sequences are available upon request). The PCR procedure was carried out in a total volume of 50 *μ*L containing 5 *μ*L of the DNA (2 ng/*μ*L), 1 *μ*L of each primer (10 *μ*M pmol), 4 *μ*L of MgCl_2_, 5 *μ*L of 10x PCR buffer, 2.5 *μ*L of dNTP (2.5 mM), 0.3 *μ*L of TaqGold DNA polymerase, and 31.2 *μ*L of H_2_O. The PCR conditions consisted of initial denaturing at 95°C for 5 min, 45 cycles at 95°C for 15 s, 54°C for 30 s, and 72°C for 15 s, and a final extension at 72°C for 5 min. The PCR products were analyzed by electrophoresis in a 2% agarose gel to confirm successful amplification. The other 40 *μ*L of PCR product was bound to streptavidin beads (GE Healthcare, Buckinghamshire, UK), purified, washed, and denatured with 0.2 mol/L NaOH solution. Then, 0.3 *μ*mol/L pyrosequencing primer was annealed to the purified single-stranded PCR product, and sequencing was done on a PyroMark ID system (Biotage, Sweden) following the manufacturer's instructions. The presence of EGFR mutations was determined by mutations in exons 18, 19, and 21.

### 2.3. Statistical Analysis

Univariate analysis of categorical variables was performed using Pearson's *χ* ^2^ test or Fisher's exact test. Multivariate logistic regression analysis with stepwise forward selection was performed to identify independent predictors for EGFR mutations. The significance of variables in the final model was evaluated after controlling for gender effect. Odds ratios (ORs) and the 95% confidence intervals (CIs) were determined. Stata (version 9.0; Stata Corp., College Station, Tex, USA) was used for statistical analyses.

## 3. Results

### 3.1. Descriptive Data

The patient characteristics are summarized in Table 1. The median age was 63 years, and 70% of the patients were men. The primary tumors ranged 1.0–10.0 cm in size (median, 4.2 cm). Sixty-five percent of patients had adenocarcinomas, and 46 patients (36%) had never smoked. The proportion of patients with stage III and IV NSCLC was 34% and 66%, respectively. Twenty-six patients (20%) presented with pleural metastases. Thirty-nine patients had stage N3 NSCLC.

### 3.2. Clinical Features Associated with EGFR Mutations

EGFR mutations were detected in 36 patients (29%), as follows: G719A (3 patients), exon 19 deletions (24 patients), and an L858R substitution in exon 21 (9 patients). Associations between clinical features and EGFR mutations were evaluated using univariate analysis; the results are listed in Table 2. Adenocarcinomas (*P* < .001), female gender (*P* < .001), and never-smokers (*P* < .001) were positively related with the presence of EGFR mutations. Tumor size, dichotomized based on the median value, was not linked with EGFR mutations (*P* = .367). Tumors with N3 nodal stage were less likely to harbor EGFR mutations (*P* = .033), whereas T stage was not related with EGFR mutations (*P* = .616). The incidence of EGFR mutations in stage IV was more common than that in stage III (*P* = .037). When distant metastatic sites (pleura, lung, liver, bone, and others) were analyzed according to EGFR mutations, pleural metastases alone were significantly associated with a high incidence of EGFR mutations (*P* = .028). Further logistic regression analysis for the patterns of pleural metastases revealed that patients with PMME showed a higher probability of EGFR mutations (OR, 7.7; 95% CI, 2.2–26.8; *P* = .001) than those without pleural metastases, whereas patients with non-PMME did not (OR, 0.6; 95% CI, 0.1–2.7; *P* = .472). On multivariate analysis, N3 nodal status and PMME, along with adenocarcinoma and a history of never-smoking, remained significant factors for EGFR mutations (Table 2). However, stage was removed as a redundant variable, and gender did not maintain a statistical significance in the final model (*P* = .805).

### 3.3. Clinical Features and TKI Responsiveness

Until July 2010, among patients who received TKI monotherapy, tumor response, as based on CT, was evaluable in 44 patients. When tumor response was classified using the Response Evaluation Criteria in Solid Tumors criteria [20], a partial response was noted in 21 patients. The response rate of 21 patients with EGFR mutations was greater than that of 23 patients with wild type (90% versus 9%, resp.; *P* < .001). Seventeen patients with N3 stage showed a tendency toward a lower response rate than 27 patients with N0–2 stage (29% versus 59%, resp.; *P* = .069). Although the response rate of 6 patients with PMME was higher than that of 38 patients without PMME (67% versus 45%, resp.), the difference was not significant (*P* = .403).

## 4. Discussion

The aim of the current study was to assess the association between metastatic presentations and EGFR mutations in stage III-IV NSCLC patients. Based on our data, patients with advanced nodal stage had a low probability of the presence of EGFR mutations compared with those with early nodal stage. In addition, we observed different presentations of pleural metastases according to EGFR mutational status. 

In this study, we focused on metastatic presentations and their associations with EGFR mutations. As in previous studies, the incidence of EGFR mutations was also within the range previously reported in [6, 7]. The predominance of EGFR mutations in never-smokers, patients with adenocarcinomas, and females was also observed [6]. Unlike prior studies, which evaluated the imaging findings of primary tumors [21, 22], our data suggested a link between metastatic sites and EGFR mutations. Although a prior study reported a high incidence of EGFR mutations in patients with brain metastases, the study was limited by a small sample size [23]. In our data, the incidence of EGFR mutations was not statistically related to metastatic sites, such as the brain and bone (Table 2). Of note, like brain and bone metastatic sites, stage alone did not maintain a significant variable regarding EGFR mutational status in the final model, whereas PMME did. Additionally, studies suggest that EGFR mutations may occur as early events in contrast to EGFR amplification [24, 25]. Thus, preferential sites of EGFR-mutant tumors, rather than tumor extent, were suggested in this study. 

The current study may offer additional insight into the spread of EGFR-mutant tumors and be helpful in the development of effective therapies. Understanding the mechanism of tumor spread may be essential in establishing effective treatment regimens. It is well known that EGFR mutations are predominantly found in patients with adenocarcinoma, history of never-smoking, and female gender [6, 7]. In this study, a strong association of EGFR mutational status with nodal stage and pattern of pleural metastases was suggested. Although the more common presentation of pleural metastases in patients with EGFR mutations was suggested in other genetic studies regarding pleural effusion [26, 27], we observed an interesting finding of the predominant presentation of PMME in EGFR-mutant tumors. Despite rare molecular data to support this finding, it is partly consistent with results of a Korean study suggesting unexpected pleural metastases at thoracotomy in females [28], one of strong predictors for EGFR mutations [6, 7]. Although it can be the case by chance and has limitation of small sample size, we believe that this finding is worthy of further molecular research. The significance of metastatic patterns also warrants further extensive studies regarding selection of beneficial patients, particularly when genetic information is unavailable.

Although the underlying mechanisms are not fully understood, vascular endothelial growth factor (VEGF) may contribute to pleural metastases with accelerating angiogenesis or lymphangiogenesis [29–31]. Laboratory findings have shown that activation of EGFR signaling may lead to VEGF expression in cancer cells [32, 33]. Interestingly, in addition to pleural metastases, different patterns of nodal metastases according to expression of the VEGF subfamily have been suggested in an animal model of lung cancer [12]. Theoretically, metastatic sites could differ by the expressed VEGF subfamily. Further molecular studies need to be conducted.

Because metastases to nodal stage were determined based on CT scans, one can question the diagnostic accuracy [34]. In clinical practice, it appears that CT alone may be a useful tool for determining the extent of disease, especially in patients with distant metastases. However, positron emission tomography, despite limited availability, warrants particular consideration because of its high accuracy of nodal stage and associations with TKI responsiveness [34, 35]. Further research needs to be conducted to understand the characteristics of patients with EGFR mutations. 

The results of this study had suggested different patterns of pleura and mediastinal nodal metastases according to the presence of EGFR mutations in patients with advanced NSCLC. This finding adds new insight into understanding the spread of EGFR-mutant tumors. However, this retrospective study needs validation with further molecular findings.

##  Conflict of Interests

The authors declare that they have no conflict of interests.

## Figures and Tables

**Table 1:** Patient characteristics (*n* = 125).

Characteristic	Number of patients (%)
Age (years)	
Median	63 (range, 28–83)
Gender	
Male	87 (70)
Female	38 (30)
Smoking history	
Never	45 (36)
Ever	80 (64)
Histopathology	
ADC*	81 (65)
Other	44 (35)
Tumor size (cm)	
Median	4.2 (range, 1.0–10.0)
T stage	
T1-2	84 (67)
T3-4	41 (33)
N stage	
N0–2	86 (69)
N3	39 (31)
M Stage	
M0	43 (34)
M1	82 (66)
Metastatic sites	
Pleura	26 (21)
PMME	13
Lung	38 (30)
Brain (*n* = 122)	23 (19)
Bone (*n* = 124)	37 (30)
Liver	7 (6)
Other^†^	14 (11)

Abbreviations: ADC, adenocarcinoma; PMME, pleural metastases with minimal effusion.

*Two bronchioloalveolar carcinomas.

^†^Including nine adrenal gland metastases.

**Table 2:** Relationship between clinical factors and EGFR mutations.

Characteristic	Number of patients with EGFR mutations (%)	*P*	OR in multivariate analysis (95% CI)	*P*
Age, years		.690	NI	
≤63	18 (27)			
>63	18 (31)			
Gender		<.001		.805
Male	15 (17)		Reference	
Female	21 (55)		0.8 (0.1–4.8)	
Smoking history		<.001		.020
Yes	12 (15)		Reference	
Never	24 (53)		7.7 (1.4–42.8)	
Histology		<.001		.005
ADC	32 (40)		9.4 (2.0–41.5)	
Others	4 (9)		Reference	
Tumor size		.367	NI	
Small (≤4.2)	21 (32)			
Large (>4.2)	15 (25)			
T stage		.616	NI	
T1-2	23 (27)			
T3-4	13 (32)			
N stage		.033		.017
N0–2	30 (35)		Reference	
N3	6 (15)		0.2 (0.0–0.7)	
M stage		.037	NI	
M0	7 (16)			
M1	29 (35)			
Metastases to pleura		.001		
PMME	10 (71)		24.2 (4.1<)	<.001
Non-PMME	2 (15)		0.8 (0.1–5.2)	.783
No	24 (24)		Reference	
Metastases to lung		.082	NI	
No	21 (24)			
Yes	15 (39)			
Metastases to brain		.075	NI	
No	24 (24)			
Yes	10 (43)			
Metastases to bone		.121	NI	
No	21 (24)			
Yes	14 (39)			
Metastases to liver		.410	NI	
No	33 (28)			
Yes	3 (43)			
Metastases to other sites		.347	NI	
No	34 (31)			
Yes	2 (14)			

Abbreviations as in Table 1.

## References

[1] Parkin DM (2001). Global cancer statistics in the year 2000. *The Lancet Oncology*.

[2] Lee CT (2002). Epidemiology of Lung Cancer in Korea. *Cancer Treatment and Research*.

[3] Wang T, Nelson RA, Bogardus A, Grannis FW (2010). Five-year lung cancer survival: which advanced stage non-small cell lung cancer patients attain long-term survival?. *Cancer*.

[4] Jemal A, Siegel R, Ward E (2006). Cancer statistics, 2006. *Ca-A Cancer Journal for Clinicians*.

[5] Sequist LV, Bell DW, Lynch TJ, Haber DA (2007). Molecular predictors of response to epidermal growth factor receptor antagonists in non-small-cell lung cancer. *Journal of Clinical Oncology*.

[6] Shigematsu H, Lin LI, Takahashi T (2005). Clinical and biological features associated with epidermal growth factor receptor gene mutations in lung cancers. *Journal of the National Cancer Institute*.

[7] Rosell R, Moran T, Queralt C (2009). Screening for epidermal growth factor receptor mutations in lung cancer. *The New England Journal of Medicine*.

[8] Detterbeck FC, Boffa DJ, Tanoue LT (2009). The new lung cancer staging system. *Chest*.

[9] Hoffman PC, Mauer AM, Vokes EE (2000). Lung cancer. *The Lancet*.

[10] Minn AJ, Gupta GP, Siegel PM (2005). Genes that mediate breast cancer metastasis to lung. *Nature*.

[11] Bos PD, Zhang XHF, Nadal C (2009). Genes that mediate breast cancer metastasis to the brain. *Nature*.

[12] Ishii H, Yazawa T, Sato H (2004). Enhancement of pleural dissemination and lymph node metastasis of intrathoracic lung cancer cells by vascular endothelial growth factors (VEGFs). *Lung Cancer*.

[13] Wu SG, Gow CH, Yu CJ (2008). Frequent epidermal growth factor receptor gene mutations in malignant pleural effusion of lung adenocarcinoma. *European Respiratory Journal*.

[14] Leung AN, Muller NL, Miller RR (1990). CT in differential diagnosis of diffuse pleural disease. *American Journal of Roentgenology*.

[15] Arenas-Jiménez J, Alonso-Charterina S, Sánchez-Payá J, Fernández-Latorre F, Gil-Sánchez S, Lloret-Llorens M (2000). Evaluation of CT findings for diagnosis of pleural effusions. *European Radiology*.

[16] Bonomo L, Feragalli B, Sacco R, Merlino B, Storto ML (2000). Malignant pleural disease. *European Journal of Radiology*.

[17] Na II, Lee TH, Choe DH (2008). A diagnostic model to detect silent brain metastases in patients with non-small cell lung cancer. *European Journal of Cancer*.

[18] Na II, Kang HJ, Cho SY (2007). EGFR mutations and human papillomavirus in squamous cell carcinoma of tongue and tonsil. *European Journal of Cancer*.

[19] Boldrini L, Gisfredi S, Ursino S (2007). Mutational analysis in cytological specimens of advanced lung adenocarcinoma: a sensitive method for molecular diagnosis. *Journal of Thoracic Oncology*.

[20] Therasse P, Arbuck SG, Eisenhauer EA (2000). New guidelines to evaluate the response to treatment in solid tumors. European Organization for Research and Treatment of Cancer, National Cancer Institute of the United States, National Cancer Institute of Canada. *Journal of the National Cancer Institute*.

[21] Glynn C, Zakowski MF, Ginsberg MS (2010). Are there imaging characteristics associated with epidermal growth factor receptor and KRAS mutations in patients with adenocarcinoma of the lung with bronchioloalveolar features?. *Journal of Thoracic Oncology*.

[22] Yano M, Sasaki H, Kobayashi Y (2006). Epidermal growth factor receptor gene mutation and computed tomographic findings in peripheral pulmonary adenocarcinoma. *Journal of Thoracic Oncology*.

[23] Matsumoto S, Takahashi K, Iwakawa R (2006). Frequent EGFR mutations in brain metastases of lung adenocarcinoma. *International Journal of Cancer*.

[24] Yatabe Y, Kosaka T, Takahashi T, Mitsudomi T (2005). EGFR mutation is specific for terminal respiratory unit type adenocarcinoma. *American Journal of Surgical Pathology*.

[25] Yatabe Y (2010). EGFR mutations and the terminal respiratory unit. *Cancer and Metastasis Reviews*.

[26] Soh J, Toyooka S, Aoe K (2006). Usefulness of EGFR mutation screening in pleural fluid to predict the clinical outcome of gefitinib treated patients with lung cancer. *International Journal of Cancer*.

[27] Kimura H, Fujiwara Y, Sone T (2006). High sensitivity detection of epidermal growth factor receptor mutations in the pleural effusion of non-small cell lung cancer patients. *Cancer Science*.

[28] Jung HH, Song KS, Park SI, Lim TH, Kui HK, Dong EG (2005). Subtle pleural metastasis without large effusion in lung cancer patients: preoperative detection on CT. *Korean Journal of Radiology*.

[29] Kraft A, Weindel K, Ochs A (1999). Vascular endothelial growth factor in the sera and effusions of patients with malignant and nonmalignant disease. *Cancer*.

[30] Kajita T, Ohta Y, Kimura K (2001). The expression of vascular endothelial growth factor C and its receptors in non-small cell lung cancer. *British Journal of Cancer*.

[31] Ferrara N, Gerber HP, LeCouter J (2003). The biology of VEGF and its receptors. *Nature Medicine*.

[32] Goldman CK, Kim J, Wong WL, King V, Brock T, Gillespie GY (1993). Epidermal growth factor stimulates vascular endothelial growth factor production by human malignant glioma cells: a model of glioblastoma multiforme pathophysiology. *Molecular Biology of the Cell*.

[33] Clarke K, Smith K, Gullick WJ, Harris AL (2001). Mutant epidermal growth factor receptor enhances induction of vascular endothelial growth factor by hypoxia and insulin-like growth factor-1 via a Pl3 kinase dependent pathway. *British Journal of Cancer*.

[34] Shim SS, Lee KS, Kim BT (2005). Non-small cell lung cancer: prospective comparison of integrated FDG PET/CT and CT alone for preoperative staging. *Radiology*.

[35] Na II, Byung HB, Hye JK (2008). ^18^F-fluoro-2-deoxy-glucose uptake predicts clinical outcome in patients with gefitinib-treated non-small cell lung cancer. *Clinical Cancer Research*.

